# Standardized patient coaching improves therapy persistence in patients with hormone receptor–positive, HER2–negative advanced/metastatic breast cancer treated with abemaciclib

**DOI:** 10.1016/j.breast.2025.104684

**Published:** 2025-12-23

**Authors:** Manfred Welslau, Peter A. Fasching, Nicole Semmler-Lins, Lothar Mueller, Erik Belleville, Lorenz Rieger, Sabrina Uhrig, Mark-Oliver Zahn, Benno Lex, Christoph Uleer, Natalija Deuerling, Tobias Hesse, Dagmar Langanke, Lothar Häberle, Hans Tesch

**Affiliations:** a Oncology Department, Klinikum Aschaffenburg, Aschaffenburg, Germany; b Department of Gynaecology and Obstetrics, Universitätsklinikum Erlangen, Friedrich-Alexander-Universität Erlangen-Nürnberg (FAU)), Erlangen, Germany; c Onkologie Untere Ems—Leer Papenburg Emden, Leer, Germany; d ClinSol GmbH & Co. KG, Wuerzburg, Germany; e Gynaecologic Oncology Department, Praxis Dr. Vehling-Kaiser, Landshut, Germany; f Oncology and Haematology Department, MVZ Onkologische Kooperation Harz, Goslar, Germany; g Department of Gynecology and Obstetrics, Kulmbach, Germany; h Gyn.-onkologische Gemeinschaftspraxis Hildesheim, Hildesheim, Germany; i Klinikum Fichtelgebirge FU, Frauenklinik, Brustzentrum Weiden - Marktredwitz, Marktredwitz, Germany; j Agaplesion Diakonieklinikum Rotenburg, Brustzentrum, Rotenburg, Germany; k St. Elisabeth Krankenhaus Leipzig, Senologie, Brustzentrum, Leipzig, Germany; l Oncology Practice at Bethanien Hospital, Frankfurt, Germany

**Keywords:** Breast cancer, Abemaciclib, Adherence, Persistence, Patient coaching, MOATT©

## Abstract

**Introduction:**

Therapy adherence is critical, particularly for patients with breast cancer undergoing oral endocrine therapies. The use of combination regimes, such as CDK4/6 inhibitors, has introduced additional side effects, which can affect adherence. A structured patient coaching and communication tool may positively affect therapy adherence.

**Methods:**

The IMPACT study (NCT04030728) was a randomized trial including patients with advanced breast cancer (aBC) receiving abemaciclib as part of routine clinical care. The study examined the influence of structured coaching on patient adherence. Participants were randomized to receive therapy management incorporating the Multinational Association of Supportive Care in Cancer (MASCC) Oral Agent Teaching Tool (MOATT©) or local standard of care practice (LSOC). The primary endpoint was the persistence rate at week 24 (PR24). Secondary endpoints included time to therapy discontinuation (TTD) and quality of life.

**Results:**

A total of 201 patients were randomized and initiated abemaciclib therapy. By week 24, 22 (10.9 %) patients had permanently discontinued abemaciclib for reasons other than progression or death: 14 (14.1 %) in the LSOC arm and 8 (7.8 %) in the MOATT© arm. PR24 was 68.9 % (95 % CI: 58.3–78.2) in the LSOC arm and 81.6 % (95 % CI: 72.5–88.7) in the MOATT© arm, yielding an odds ratio of 2.01 (95 % CI: 1.02–3.96; *P* = 0.04). TTD showed a hazard ratio of 0.59 (95 % CI: 0.32–1.07), favoring the MOATT© arm. No significant differences in quality of life between study arms were observed.

**Conclusion:**

Individual patient coaching based on MOATT© demonstrated improved PR24 for patients undergoing abemaciclib treatment for aBC.

## Highlights

• Premature therapy discontinuation is a key challenge in breast cancer management.
• In the control group, 14.1 % of patients discontinued abemaciclib prematurely.
• Individual patient coaching improved 24-week persistence under abemaciclib therapy.

## 1 Introduction

Endocrine therapies are the cornerstone in the treatment of hormone receptor-positive/human epidermal growth factor receptor 2-negative (HRpos/HER2neg) breast cancer. Both in early-stage and advanced-stage settings, these therapies have substantially improved patient survival rates [[1], [2], [3], [4], [5], [6]]. The therapeutic landscape of HRpos/HER2neg advanced breast cancer (aBC) has changed, particularly with the introduction of cyclin-dependent kinase 4/6 (CDK4/6) inhibitors. Palbociclib, ribociclib, and abemaciclib were approved between 2015 and 2017 for use in combination with endocrine therapy [[7], [8], [9]]. Their approval was based on multiple randomized controlled trials showing a significant improvement in progression-free survival (PFS) compared with endocrine therapy alone. Owing to their proven efficacy, CDK4/6 inhibitors combined with endocrine therapy are now considered the standard first-line treatment for patients with HRpos/HER2neg aBC [10]. Furthermore, studies suggest that chemotherapy, historically administered to this subgroup of patients, does not offer a survival benefit compared to CDK4/6 inhibitor therapy when combined with endocrine therapy in the first or second-line setting [11]. However, concerns regarding treatment adherence and compliance with oral cancer therapies were raised early on [[12], [13], [14], [15]]. According to the World Health Organization (WHO), adherence is defined as the extent to which a person's behavior – taking medication, following a diet, or executing lifestyle changes-corresponds with agreed recommendations from a health care provider [16,17]. A large adjuvant trial showed that non-adherence is linked to reduced disease-free survival [18]. This correlation underscores the critical need to understand and improve therapy management for these patients.

In the metastatic setting, adherence remains a crucial issue. The EvAluate-TM study indicated that over 22 % of patients with aBC receiving endocrine monotherapy with letrozole discontinue treatment within 12 months of starting for reasons other than disease progression [19]. The introduction of CDK4/6 inhibitors has brought adherence to the forefront of clinical discussions. Prior to these inhibitors, 40 %–50 % of all patients with HRpos/HER2neg disease were treated with chemotherapy in the first-line setting [20]. With the introduction of CDK4/6 inhibitors and trials showing improved survival compared to standard chemotherapy [21], these inhibitors have become the standard in first-line treatment in 70 %–80 % of cases [20]. Adherence issues, however, are also evident in patients treated with this class of drugs. Across pivotal first-line trials of CDK4/6 inhibitors, discontinuation due to adverse events occurred in 7.5 % of patients treated with ribociclib (MONALEESA-2), 9.3 % with palbociclib (PALOMA-2), and 19.6 % with abemaciclib (MONARCH 3) [7,22,23]. Furthermore, it was shown that adherence to CDK4/6 inhibitor therapy is associated with better overall survival (HR: 0.393, 95 % CI: 0.188–0.822; P < 0.0131) and progression-free survival (HR: 0.408, 95 % CI: 0.222–0.750; P < 0.0039) [24]. In general, a longer duration of CDK4/6 inhibitor therapy and an older age were associated with better overall (HR, 0.55; 95 % CI, 0.41–0.73; P < 0.001) or progression-free survival (HR, 0.99; 95 % CI 0.98–1.00; P = 0.03), respectively [25]. A dose reduction was associated with shorter treatment duration and reduced overall survival (39.9 versus 54.3 months) [26]. Thus, adherence is a major concern for patients treated with CDK4/6 inhibitors and endocrine therapy in the metastatic setting.

Therefore, the IMPACT study aimed to examine whether the standardized Multinational Association of Supportive Care in Cancer (MASCC) Oral Agent Teaching Tool (MOATT©) can improve adherence in patients treated with abemaciclib and endocrine therapy for aBC. Abemaciclib was selected for this study because adverse events during treatment resulted in higher discontinuation events when comparing the pivotal first-line trials [7,22,23]. Furthermore, unlike palbociclib and ribociclib, it is administered continuously without treatment-free intervals and has a distinct toxicity profile with more gastrointestinal side effects, which may particularly influence adherence patterns [27].

MOATT© is a patient education tool specifically developed for oncology healthcare providers in clinical practice to guide patient education on oral anticancer therapies. It aims to guide patient teaching, provide comprehensive, standardized instruction, and encourage adherence to oral chemotherapy treatment [28,29]. It has been validated in some clinical settings, primarily focusing on oral chemotherapy [30]. However, there is limited data on the effect of MOATT© on the adherence to oral cancer therapies with a more favorable side-effect profile, such as CDK4/6 inhibitors. Therefore, the IMPACT study aimed to evaluate the influence of standardized patient education and coaching on persistence rates, side effect management, and unplanned therapy interruptions in patients treated with abemaciclib and endocrine therapy.

## 2 Methods

### 2.1 Study design and conduct

The Implementing Patients’ Competence in Oral Breast Cancer Therapy study (IMPACT; NCT04030728) is a prospectively randomized and controlled non-interventional study that allocated patients in a 1:1 ratio to one of two study arms.

• Patient management according to local standard of care practice (LSOC arm) or
• Standardized MOATT© patient coaching (MOATT© arm).

Patients with advanced or metastatic breast cancer proven by clinical measures (i. e. standard imaging) were eligible if they had an indication for abemaciclib treatment as specified in the Summary of Product Characteristics (SmPC) [31]. Patients experiencing a visceral crisis were excluded. Complete inclusion and exclusion criteria are listed in Table S1, and the study design is illustrated in Fig. S1.

The study was carried out in accordance with the Code of Ethics of the World Medical Association (Declaration of Helsinki) and all procedures were conducted in compliance with all laws and regulations, as well as established guidelines and recommendations. Ethical approval was provided by the Ethics Committee of the Bavarian Medical Association, München, Germany (ethical approval number 19046; December 17th, 2019) and the appropriate ethics committees or institutional review boards at all sites, and all participants provided written informed consent. Recruitment occurred across 25 study sites in Germany between June 2020 and February 2022. The database was last updated on May 3, 2023.

### 2.2 MOATT© coaching and local standard of care practice

At each study site, staff received training to provide MOATT©-based coaching. Study site staff participated in a dedicated workshop and received the MOATT© User Guide, which included detailed instructions and standardized examples for patient counseling. A practical demonstration of the MOATT© platform and its application during patient interactions was conducted by the study team. Participation in the OnkoCoach training program (Arbeitskreis Klinische Studien e. V.), which provides structured education for non-physician staff in oncological patient counseling, was recommended but not mandatory. The MOATT©-based coaching comprises four sections. The first section assesses the patient's understanding of their treatment plan, current medications, and their capability to manage an oral cancer agent. The second section offers general instructions for all oral cancer agents, including storage, handling, disposal, and strategies for remembering to take the medication. It also outlines steps for various scenarios, such as missed doses. The third section provides specific information about the drug, including dosage, schedule, side effects, and potential interactions. The final section contains questions to confirm the patient's understanding of the provided information [28]. Patients in the MOATT© arm received this coaching prior to starting abemaciclib treatment and at weeks 2, 6, 12, 18, and 24 thereafter. Examples of the MOATT© are available in different languages through the Multinational Association for Supportive Care in Cancer (MASCC; www.mascc.org) website.

Patients in the LSOC arm received information about the treatment according to LSOC without additional standardized instructions.

### 2.3 Treatment

Patients received orally administered abemaciclib (Verzenios®, Lilly Deutschland GmbH), either combined with an aromatase inhibitor or fulvestrant as initial endocrine-based therapy or following prior endocrine therapy. All treatments were prescribed and administered in accordance with the medical practices of each center, and treatment adjustments were made at the discretion of the treating physician.

### 2.4 Objectives and endpoints

The primary objective was to compare the influence of patient management according to routine local practices versus continuous standardized patient education and coaching using the MOATT© on the persistence rate. Persistence rate within this study was defined as the proportion of patients who were still under abemaciclib treatment at week 24 (PR24) without a permanent termination of therapy. Persistence is the duration of time from initiation to discontinuation of therapy [32]. In contrast, adherence refers to the degree or extent of conformity to the recommendations about day-to-day treatment by the provider with respect to the timing, dosage, and frequency [32]. Adherence was not assessed within the trial. Secondary objectives included assessing therapy interruptions and quality of life. PFS and side effects, as assessed during clinical routine, were also documented. PFS was included to confirm that the educational intervention would not negatively affect treatment efficacy.

### 2.5 Statistical considerations

Sample size calculations for this study were based on the assumption that the MOATT©-based approach would increase the PR24 from 75 % to 90 %. A significance level α = 0.05 and a power of 80 % were considered. Sample size calculations yielded a total of 200 patients and a dropout rate of 5 % was expected.

The difference in persistence rates was evaluated using an odds ratio with a corresponding 95 % confidence interval (CI). To determine the presence of a statistically significant difference in PR24 between the randomization arms, a two-sided χ^2^-test was employed, with the significance level set at α = 0.05.

The secondary objectives were evaluated using descriptive statistical methods. For continuous and ordinal endpoints, summaries were provided in the form of means and standard deviations for each randomization arm. Similarly, categorical endpoints were detailed as frequencies and percentages for each arm.

The PFS was defined as the interval from the date of randomization to either the onset of disease progression, confirmed via clinical measures as per expert assessments and routine clinical practices, or death from any cause. The PFS rates at week 24 were estimated for each randomization arm using the Kaplan–Meier product limit method, and 95 % CIs were presented.

Persistence time, which was defined as the interval from the date of randomization until either the interruption of therapy or the end of therapy for any reason, was censored at week 26 of therapy. The persistence rate at week 24 was calculated for each randomization arm using the Kaplan–Meier method, with 95 % CIs displayed. Additionally, persistence time across both treatment arms was analyzed using a simple Cox regression model with the treatment arm as the sole predictor and the resulting hazard ratio with a 95 % CI was shown.

Quality of life was assessed at weeks 2, 6, 12, 18, and 24 using the Functional Assessment of Cancer Therapy - Breast (FACT-B) total score and the National Comprehensive Cancer Network (NCCN) Distress Thermometer score. The FACT-B score is comprised of subscores for physical well-being (PWS), functional well-being (FWB), breast cancer subscale (BCS), social well-being (SWB), and emotional well-being (EWB). A summary of the statistics for all subscores and the overall total score was provided. For the NCCN Distress Thermometer, a cutoff value of 3 was selected to identify patients with clinically substantial levels of distress.

The duration of coaching was documented for each visit for patients in the MOATT© arm.

All adverse events were coded in accordance with the NCI Common Terminology Criteria for Adverse Events (CTCAE) version 5.0. In assessing the incidence of adverse events, along with any sub-classifications by treatment, time period, and severity, each participant was counted once, and repetitions of adverse events were disregarded; the denominator was the total population size. The overall incidence was then calculated.

All statistical calculations were conducted using the R system for statistical computing (version 4.2.1; R Development Core Team, Vienna, Austria, 2022).

## 3 Results

### 3.1 Patient disposition

**Fig. 1:**
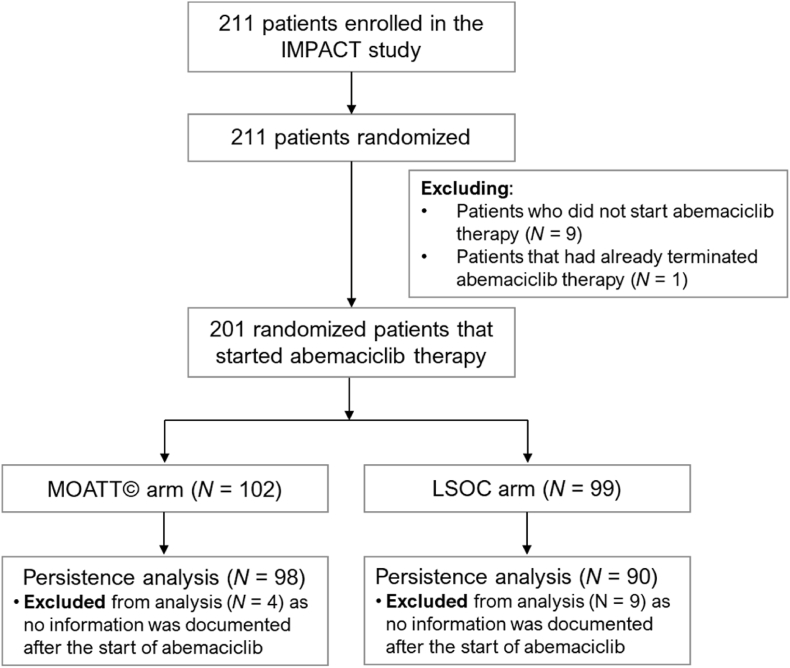
Patient flow chart. *MOATT©: Multinational Association of Supportive Care in Cancer (MASCC) Oral Agent Teaching Tool; LSOC: local standard of care*.

A total of 211 patients were included in the IMPACT study. Of these, 9 did not start abemaciclib therapy and were therefore excluded from the analysis. One additional patient was excluded due to implausible data, as they were enrolled after abemaciclib therapy had already been terminated. Consequently, the patient population consisted of 201 individuals. Patient disposition is shown in Fig. 1. After enrolment, patients were randomized into two study arms. The LSOC arm included 99 patients, while the MOATT© arm comprised 102 patients. For the persistence analysis, 13 patients (4 in the MOATT© arm and 9 in the LSOC arm) were excluded because no information was documented after the start of abemaciclib therapy (Fig. 1). For the presentation of safety events and the time-to-therapy-discontinuation, PFS and quality of life analyses, all patients were included to provide a comprehensive overview of therapy exposure and to avoid bias within the time-to-event framework.

### 3.2 Patient characteristics

The patient characteristics are listed in Table 1. The majority of the patients, 131 (66.2 %), were treated in the first-line setting. First-line therapy aBC is defined as the first systemic treatment administered after diagnosis of advanced/metastatic disease. Metastases were most frequently observed in visceral organs (*N* = 89; 48.9 %). Bone-only disease was described in 30.2 % of the patients (*N* = 55). Most patients had not been previously treated with chemotherapy (*N* = 117; 58.2 %). The most common combination partner was letrozole (*N* = 114; 57.6 %), followed by fulvestrant, which was administered to 56 patients (28.3 %) (Table 2).

**Table 1:** Patient characteristics by treatment arm.

Characteristic	Level	*N* (%) or mean (SD)		
		Total (*N* = 201)	MOATT© (*N* = 102)	LSOC (*N* = 99)
Age (year)	–	65.5 (14.1)	63.7 (13.0)	67.4 (15.0)
BMI (kg/m^2^)	–	26.9 (6.0)	27.0 (5.8)	26.9 (6.1)
ECOG	0	109 (57.7)	54 (57.4)	55 (57.9)
	1	68 (36.0)	34 (36.2)	34 (35.8)
	≥2	12 (6.3)	6 (6.4)	6 (6.3)
	Missing	12	8	4
ER status	ER+	171 (98.3)	85 (97.7)	86 (98.9)
	ER−	3 (1.7)	2 (2.3)	1 (1.1)
	Missing	27	15	12
PgR status	PgR+	142 (81.6)	73 (83.0)	69 (80.2)
	PgR−	32 (18.4)	15 (17.0)	17 (19.8)
	Missing	27	14	13
Tumor grading	G1/2	100 (63.3)	52 (65.0)	48 (51.5)
	G3	58 (36.7)	28 (35.0)	30 (38.5)
	Missing	43	22	21
Metastases pattern	Brain	3 (1.6)	0 (0.0)	3 (3.3)
	Visceral	89 (48.9)	46 (50.0)	43 (47.8)
	Bone	55 (30.2)	28 (30.4)	27 (30.0)
	Others	35 (19.2)	18 (19.6)	17 (18.9)
	Missing	19	10	9
Therapy line	1st line	131 (66.2)	62 (61.4)	69 (71.1)
	2nd or higher line	67 (33.8)	39 (38.6)	28 (28.9)
	Missing	3	1	2
Previous chemotherapy	Yes	84 (41.8)	44 (43.1)	40 (40.4)
	No	117 (58.2)	58 (56.9)	59 (59.6)
Previous endocrine therapy	Yes	83 (41.3)	49 (48.0)	34 (34.3)
	No	118 (58.7)	53 (52.0)	65 (65.7)
Previous radiotherapy	Yes	91 (45.3)	52 (51.0)	39 (39.4)
	No	110 (54.7)	50 (49.0)	60 (60.6)
Concomitant diseases	1	47 (30.5)	25 (32.9)	22 (28.2)
	2	37 (24.0)	22 (28.9)	15 (19.2)
	3	29 (18.8)	9 (11.8)	20 (25.6)
	4+	41 (26.6)	20 (26.3)	21 (26.9)
	Missing	47	26	21

MOATT©: Multinational Association of Supportive Care in Cancer (MASCC) Oral Agent Teaching Tool; LSOC: local standard of care; BMI: body mass index; ER: estrogen receptor; PgR: progesterone receptor; SD: standard deviation.

**Table 2:** Therapy situation and endocrine combination partner for abemaciclib.

Treatment and therapy situation		*N* (%)		
		Total (*N* = 201)	MOATT© (*N* = 102)	LSOC (*N* = 99)
Therapy situation	First line therapy	124 (62.6)	57 (56.4)	67 (69.1)
	Progress in the advanced situation	67 (33.8)	39 (38.6)	28 (28.9)
	Toxicity in previous therapy line	4 (2.0)	4 (4.0)	0 (0.0)
	Other	3 (1.5)	1 (1.0)	2 (2.1)
	Missing	3	1	2
Endocrine combination partner	Letrozole	114 (57.6)	57 (56.4)	57 (58.8)
	Anastrozole	4 (2.0)	2 (2.0)	2 (2.1)
	Exemestane	22 (11.1)	11 (10.9)	11 (11.3)
	Fulvestrant	56 (28.3)	30 (29.7)	26 (26.8)
	Other	2 (1.0)	1 (1.0)	1 (1.0)
	Missing	3	1	2

MOATT©: Multinational Association of Supportive Care in Cancer (MASCC) Oral Agent Teaching Tool; LSOC: local standard of care.

### 3.3 Persistence

At the end of the study, at week 24, 142 patients remained on treatment, with 80 patients (78.4 %) in the MOATT© arm and 62 patients (62.6 %) in the LSOC arm (Table 3). Overall, 22 out of all patients (10.9 %) permanently discontinued the treatment before progress or death; 14 in the LSOC arm (14.1 %) and 8 (7.8 %) in the MOATT© arm. In the LSOC arm, discontinuations were due to toxicity (N = 7), patient choice (N = 6), and other reasons (N = 1). In the MOATT© arm, 3 discontinued abemaxiclib due to toxicity and 5 by patient choice (Table 3).

**Table 3:** Distribution of reasons for study termination at week 24.

Reason for termination of the study	*N* (%)		
	Total (*N* = 201)	MOATT© (*N* = 102)	LSOC (*N* = 99)
Terminated before week 24 (all reasons)	59 (29.4)	22 (21.6)	37 (37.4)
Regular end of study (after 24 weeks)	142 (70.6)	80 (78.4)	62 (62.6)
Reasons for termination before week 24:			
Death	8 (4.0)	4 (3.9)	4 (4.0)
Withdrawal of consent	7 (3.5)	2 (2.0)	5 (5.1)
Lost to follow-up	6 (3.0)	2 (2.0)	4 (4.0)
Progressive disease	16 (8.0)	6 (5.9)	10 (10.1)
Permanent discontinuation of abemaciclib	22 (10.9)	8 (7.8)	14 (14.1)
Participation in another clinical trial with IMP	0 (0.0)	0 (0.0)	0 (0.0)

MOATT©: Multinational Association of Supportive Care in Cancer (MASCC) Oral Agent Teaching Tool; LSOC: local standard of care.

**Fig. 2:**
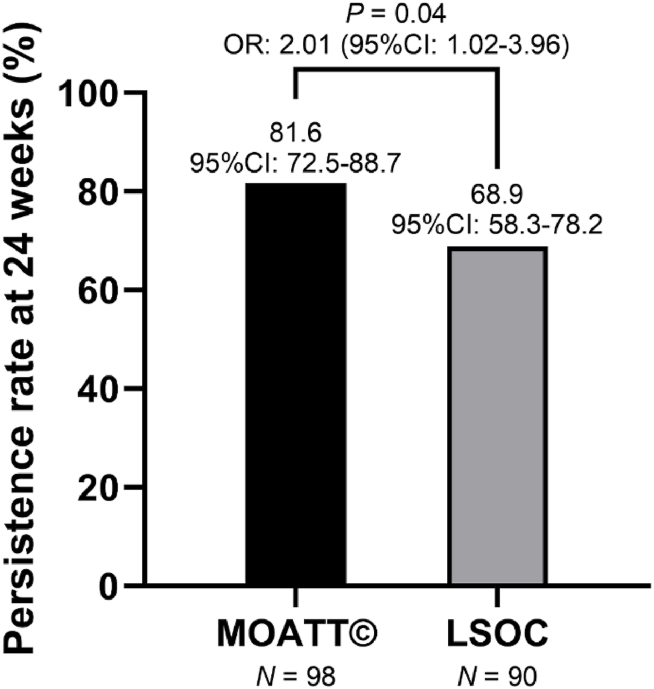
Persistence rate at week 24. *MOATT©: Multinational Association of Supportive Care in Cancer (MASCC) Oral Agent Teaching Tool; LSOC: local standard of care; OR: odds ratio*.

The PR24 was 68.9 % (95 % CI: 58.3–78.2) in the LSOC arm and 81.6 % (95 % CI: 72.5–88.7) in the MOATT© arm. This corresponded to an odds ratio of 2.01 (95 % CI: 1.02–3.96, *P* = 0.04) favoring the MOATT© arm (Fig. 2).

**Fig. 3:**
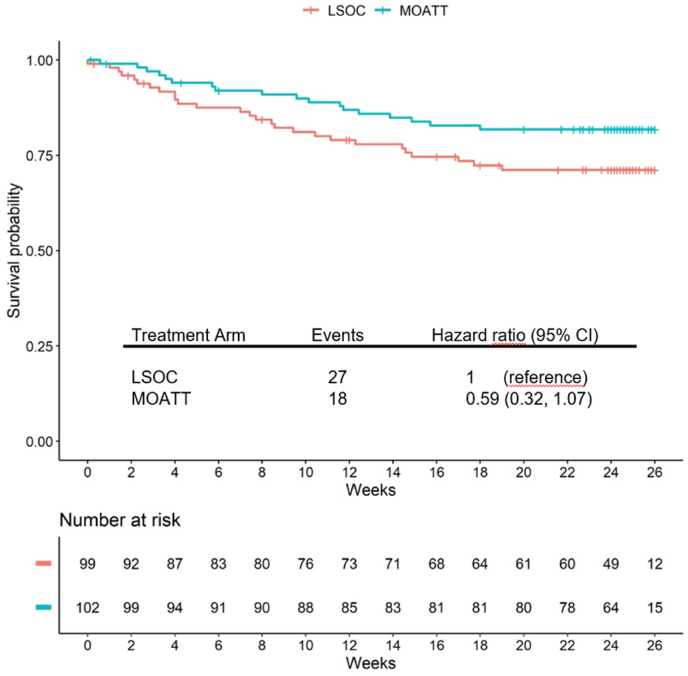
Time to therapy discontinuation for both randomization arms. *MOATT©: Multinational Association of Supportive Care in Cancer (MASCC) Oral Agent Teaching Tool; LSOC: local standard of care*.

**Fig. 4:**
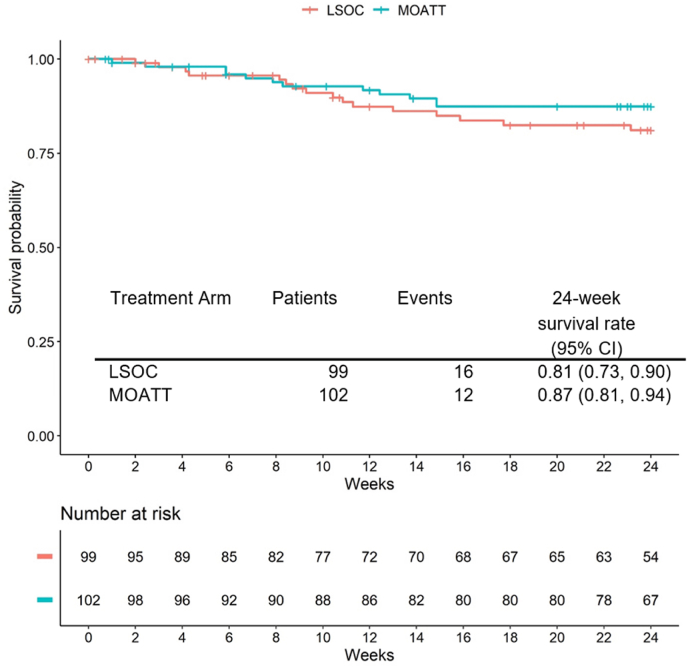
Progression-free survival according to randomization arms. *MOATT©: Multinational Association of Supportive Care in Cancer (MASCC) Oral Agent Teaching Tool; LSOC: local standard of care*.

Additionally, a sensitivity analysis involving a survival analysis with time to permanent discontinuation of abemaciclib therapy was conducted. The hazard ratio favoring the MOATT© arm was 0.59 (95 % CI: 0.32–1.07) (Fig. 3). PFS was comparable between the randomization arms (Fig. 4).

### 3.4 Therapy interruptions

Therapy interruptions were reported for 65 patients (32.3 %), with 35 in the MOATT© arm (34.3 %) and 30 in the LSOC arm (30.3 %). The mean cumulative duration of therapy interruption was 3.4 days (±11.9) in the MOATT© arm and 2.5 days (±11.1) in the LSOC arm. The total mean duration of therapy was longer in the MOATT© arm (139.9 ± 51.4 days) than in the LSOC arm (128.6 ± 57.4 days). Reasoning for therapy interruptions was not assessed.

### 3.5 Quality of life

Quality of life and the NCCN distress thermometer were assessed at each study visit (Table 4). There were no substantial differences in the main outcome measures, FACT-B TOI, or FACT-B total scores. Similarly, there were no differences in the FACT subscales (Table 4).

**Table 4:** Quality of life assessment with FACT-B questionnaire and NCCN distress thermometer.

Subset	Arm	Mean (SD)					
		Baseline	Week 2	Week 6	Week 12	Week 18	Week 24
FACT-B TOI	MOATT©	62.9 (15.3)	64.6 (14.5)	62.9 (15.0)	62.2 (15.7)	64.1 (14.3)	65.8 (14.6)
	LSOC	61.6 (17.3)	60.9 (16.8)	63.1 (17.0)	63.0 (18.5)	65.0 (16.6)	64.1 (16.8)
FACT-B total score	MOATT©	100.6 (20.6)	102.9 (19.9)	100.6 (20.7)	100.1 (21.2)	101.8 (20.1)	104.5 (21.1)
	LSOC	99.8 (21.6)	99.3 (21.2)	103.4 (22.2)	102.2 (25.1)	104.6 (22.3)	103.0 (22.6)
Physical well-being	MOATT©	20.2 (6.0)	19.7 (6.1)	19.3 (6.4)	19.1 (6.7)	19.6 (6.0)	20.5 (5.3)
	LSOC	19.4 (6.7)	17.4 (7.6)	18.8 (7.1)	18.9 (7.3)	20.1 (5.9)	20.3 (6.2)
Social well-being	MOATT©	22.0 (5.4)	22.1 (5.0)	21.7 (5.4)	21.4 (5.6)	21.6 (5.4)	21.6 (5.5)
	LSOC	23.0 (4.9)	22.6 (5.3)	23.2 (4.9)	22.9 (5.5)	22.7 (5.4)	21.9 (5.7)
Emotional well-being	MOATT©	15.5 (5.1)	16.4 (4.9)	16.2 (5.0)	16.1 (5.2)	15.8 (5.1)	17.1 (4.6)
	LSOC	15.0 (5.0)	15.3 (5.2)	16.6 (5.2)	16.2 (5.7)	16.5 (5.0)	16.6 (4.6)
Functional well-being	MOATT©	15.6 (6.7)	15.8 (6.3)	15.3 (6.4)	15.7 (6.1)	16.4 (5.4)	17.4 (5.7)
	LSOC	15.6 (6.7)	15.3 (6.3)	16.3 (6.4)	16.0 (7.0)	16.6 (6.9)	17.2 (6.1)
Breast cancer subscale	MOATT©	27.0 (5.4)	28.7 (4.9)	28.2 (5.2)	27.7 (5.4)	27.2 (5.5)	27.3 (5.7)
	LSOC	27.2 (6.3)	27.8 (5.7)	28.3 (5.8)	27.9 (6.7)	28.1 (6.0)	26.9 (6.4)
NCCN distress thermometer	MOATT©	5.7 (2.5)	4.6 (2.4)	5.1 (2.5)	5.5 (2.4)	5.1 (2.5)	4.4 (2.4)
	LSOC	5.4 (2.5)	5.5 (2.6)	4.9 (2.6)	4.9 (2.6)	4.6 (2.4)	4.6 (2.6)

SD: standard deviation; MOATT©: Multinational Association of Supportive Care in Cancer (MASCC) Oral Agent Teaching Tool; LSOC: local standard of care.

### 3.6 Safety analysis

Adverse events of all grades occurred frequently in both therapy management groups, with 93.1 % of patients in the MOATT© arm and 92.9 % in the LSOC arm, as detailed in Table 5. The MOATT© approach resulted in severe adverse events in 15.7 % of patients, compared with 21.2 % of patients receiving LSOC.

**Table 5:** Overview of the safety events.

	MOATT© (*N* = 102 patients)		LSOC (*N* = 99 patients)		Total (*N* = 201 patients)	
	Events	Patients *N* (%)	Events	Patients *N* (%)	Events	Patients *N* (%)
**AEs**	665	95 (93.1)	671	92 (92.9)	1336	187 (93.0)
**Ars**	401	93 (91.2)	470	89 (89.9)	871	182 (90.5)
**SAEs**	29	16 (15.7)	30	21 (21.2)	59	37 (18.4)
**SARs**	8	6 (5.9)	10	7 (7.1)	18	13 (6.5)
**Fatal events**	4	4 (3.9)	4	4 (4.0)	8	8 (4.0)
**Fatal reactions**	1	1 (1.0)	1	1 (1.0)	2	2 (1.0)

MOATT©: Multinational Association of Supportive Care in Cancer (MASCC) Oral Agent Teaching Tool; LSOC: local standard of care; AEs: adverse events; ARs: adverse reactions; SAEs: serious adverse events; SARs: serious adverse reactions.

Adverse events were graded according to the NCI CTCAE, version 5.0. The most common adverse events (all grades) were diarrhea, fatigue, nausea, pain, and vomiting. The frequency of these events did not show substantial differences between the two therapy management approaches. Anemia was the most prevalent hematologic side effect, affecting 10 % of patients (*N* = 10) in the MOATT© arm and 5.1 % (*N* = 5) in the LSOC arm. Grade 3/4 events occurred rarely. Diarrhea, nausea and white blood cell decrease were the most common grade 3/4 events affecting 4 % (N = 4) and 1 % (N = 1), 1 % (N = 1) and 3 % (N = 3), and 3 % (N = 3) and 1 % (N = 1) of the MOATT© and LSOC arm, respectively. The most common side effects are listed in Table 6.

**Table 6:** Adverse events according to randomization arm and NCI CTCAE Version 5.0 terminology.

CTCAE version 5.0 terminology	All Grade	Grade 3/4		
	MOATT©	LSOC	MOATT©	LSOC
	*N*	*N*	*N*	N
Diarrhea	66 (65)	56 (57)	4 (4)	1 (1)
Fatigue	47 (46)	38 (38)	0 (0)	0 (0)
Nausea	35 (34)	37 (37)	1 (1)	3 (3)
Other gastrointestinal disorders	24 (24)	38 (38)	0 (0)	1 (1)
Pain	23 (23)	20 (20)	1 (1)	1 (1)
Vomiting	11 (11)	21 (21)	0 (0)	2 (2)
Anemia	10 (10)	5 (5.1)	1 (1)	0 (0)
Back pain	7 (7)	10 (10)	0 (0)	1 (1)
Bone pain	7 (7)	1 (1)		
Constipation	7 (7)	3 (3)	0 (0)	0 (0)
Alopecia	6 (6)	3 (3)	0 (0)	0 (0)
Anorexia	6 (6)	5 (5.1)	0 (0)	0 (0)
Other blood and lymphatic system disorders	6 (6)	9 (9.1)	1 (1)	2 (2)
Dyspnea	6 (6)	6 (6.1)	1 (1)	1 (1)
Hot flashes	6 (6)	1 (1)	0 (0)	0 (0)
Weight loss	6 (6)	1 (1)	0 (0)	0 (0)
Abdominal pain	5 (5)	2 (2)	0 (0)	0 (0)
Headache	5 (5)	2 (2)	0 (0)	0 (0)
Other hepatobiliary disorders	5 (5)	4 (4)	0 (0)	1 (1)
Dysgeusia	4 (4)	4 (4)	0 (0)	0 (0)
Fever	4 (4)	2 (2)	0 (0)	0 (0)
Other respiratory, thoracic, and mediastinal disorders	4 (4)	2 (2)	1 (1)	0 (0)
Stomach pain	4 (4)	0 (0)	0 (0)	0 (0)
Neutrophil count decreased	3 (3)	5 (5.1)	1 (1)	1 (1)
Pain in extremity	3 (3)	3 (3)	0 (0)	0 (0)
White blood cells decreased	3 (3)	4 (4)	3 (3)	2 (2)
Pruritus	2 (2)	5 (5.1)	0 (0)	0 (0)
Vertigo	2 (2)	5 (5.1)	0 (0)	0 (0)
Cough	1 (1)	3 (3)	0 (0)	0 (0)
Dizziness	1 (1)	5 (5.1)	0 (0)	0 (0)
Dyspepsia	1 (1)	5 (5.1)	0 (0)	1 (1)
Insomnia	1 (1)	5 (5.1)	0 (0)	0 (0)
Other nervous system disorders	1 (1)	4 (4)	0 (0)	0 (0)
Premature menopause	0 (0)	3 (3)	0 (0)	0 (0)
Other vascular disorders	0 (0)	3 (3)	0 (0)	1 (1)
Febrile neutropenia			1 (1)	1 (1)

MOATT©: Multinational Association of Supportive Care in Cancer (MASCC) Oral Agent Teaching Tool; LSOC: local standard of care; CTCAE: NCI Common Terminology Criteria for Adverse Events.

## 4 Discussion

In the randomized IMPACT study, we demonstrated that a MOATT©-based coaching program can improve adherence to therapy in patients treated with abemaciclib and endocrine therapy. The persistence rate for patients who underwent coaching by MOATT© was significantly higher compared to the population who received standard of care for follow-up (81.6 and 68.9 %, respectively). Furthermore, 14.1 % of patients in the control arm terminated therapy prematurely, which underscores the significance of this issue in the management of patients with advanced HRpos/HER2neg breast cancer.

Data on adherence to the combination of CDK4/6 inhibitors and endocrine therapy in patients with aBC remains limited. In previous randomized trials with abemaciclib, palbociclib, and ribociclib, 9.3 %–19.6 % of patients discontinued treatment over median follow-up periods of 15.3–23 months [7,22,23]. In our study, 10.9 % of patients discontinued abemaciclib within 24 weeks, which is consistent with previously reported rates. Notably, this rate was even higher in the LSOC arm (14.1 %) than in the MOATT©arm (7.8 %), suggesting that structured patient education and therapy management can substantially influence patient adherence in this context.

MOATT© is a standardized program designed to guide primarily healthcare professionals in conducting detailed, personalized interactions with patients. Notably, both physicians and breast care nurses can use the tool, which might help to reduce the oncologist's workload. Although this approach improved adherence, the quality-of-life metrics did not differ between the two therapy management approaches. Given resource constraints, the integration of digital tools to complement patient interactions has been increasingly discussed. For example, the PreCycle study implemented a digital tool serving as a treatment support service that included daily drug intake documentation, daily general health assessments, symptom tracking, feedback functions, and quality-of-life documentation [33,34]. Within the randomized study, the comparison of the active digital tool with a version with limited functionality showed that its use was associated with better maintenance of quality of life [34]. Over an observation period of approximately 18–20 months, the use of the digital tool resulted in fewer quality-of-life deteriorations—a difference of 9.1 % (35.1 % in the arm with the digital tool vs. 44.2 % in the control arm). However, the study also noted a 7.3 % increase in therapy discontinuations in the arm using the active digital tool [34]. This highlights that both in-person patient education as well as digital support tools might be important to improve both quality of life and therapy adherence.

A study focusing on endocrine monotherapy in the metastatic setting identified predictors for non-adherence [19]. Time to therapy discontinuation was notably shorter in patients experiencing more adverse events (hazard ratio = 8.2; 95 % CI: 3.0–22.5) and in those who, at the start of therapy, reported a general non-compliance with treatments in the last 30 days (hazard ratio = 2.79; 95 % CI: 1.3–6.0) or expressed a likelihood of omitting drug intake when feeling unwell (hazard ratio = 4.0; 95 % CI: 1.9–8.4). These findings highlight the substantial impact of side effects and individual patient characteristics on adherence to oral anticancer therapies in aBC and emphasize the need for supportive tools specifically tailored to support such subpopulations.

In the future, integrating a personal therapy management approach such as MOATT© possibly in combination with a digital tool, may enhance both quality of life and adherence. The IMPACT trial underscores the ongoing medical need to improve therapy management for patients treated with CDK4/6 inhibitors and endocrine treatments. Even though the presented data focus on aBC, extending this approach to early breast cancer in future studies and routine care will be essential in light of recent trials and treatment guideline updates integrating CDK4/6 inhibitors into the adjuvant setting [5,6,35].

In contrast to the metastatic setting, data on persistence, adherence, and patient compliance have been extensively studied in the early breast cancer context. For these patients, issues with non-adherence to both therapy and follow-up care programs have been noted. Notably, about 20 % of early breast cancer patients do not return for routine follow-up care mammograms [36], highlighting a substantial gap in maintaining regular patient contact. Existing research, including older studies on aromatase inhibitors and tamoxifen, as well as more recent studies on CDK4/6 inhibitors [[37], [38], [39], [40], [41], [42]], reports 5-year adherence rates at 66.2 % and persistence rates at 66.8 % [37]. These figures underscore the importance of adherence and persistence, particularly as they relate to prognosis deterioration [18]. Concerning new combination therapies such as abemaciclib with endocrine treatment and ribociclib with aromatase inhibitors in the early therapy setting, about 20 % of patients discontinue treatments prematurely due to side effects [[43], [44], [45], [46]]. This suggests that the findings from the IMPACT study could be relevant for further investigation in the adjuvant setting.

With the increasing use of CDK4/6-Inhibitors in the adjuvant setting, an improved management of side effects will be critical for managing these treatment approaches [47,48]. This, however, could result in a substantial patient-management workload over a treatment period of 2–3 years. Incorporating MOATT© and potentially a digital solution could play a crucial role in reshaping the future of therapy management for these patients.

Coaching of medical staff and personalized teaching of patients, however, is also a time-consuming procedure and, thus, approaches that are not in the need of face-to-face interactions might be of interest. That such non-interactive, non-personalized approaches are not the perfect solution to improve therapy management in the adjuvant setting was shown in a comprehensive randomized study. In this study, patients received extensive information by mail during the first year of therapy, along with monthly reminders about maintaining endocrine therapy. This information was designed to address relevant issues at specific therapy stages and was developed in close collaboration with breast cancer survivors. Despite these efforts, the control group, which did not receive this informational material [38], showed only a slight difference in persistence to anastrozole treatment compared to the educational arm (43 % in the educational arm vs. 40.5 % in the control arm) [38]. This underscores that face-to-face patient coaching, as demonstrated in the IMPACT trial, may offer greater benefits than non-interactive approaches.

While the randomized IMPACT study met its primary endpoint, it has several limitations that need to be noted. First, the sample size was limited, with only 22 patients prematurely discontinuing treatment. Although the primary endpoint was statistically significant, the time to treatment discontinuation did not achieve statistical significance, with a hazard ratio of 0.59 and a 95 % CI of 0.32–1.07. Additionally, the observation period of 24 weeks was shorter than in other studies in the aBC setting that provide adherence or persistence data, limiting the comparability with those studies. Nonetheless, IMPACT, as a randomized study, contributes to the evidence on improving adherence through a personalized healthcare approach.

In conclusion, this study is one of the first in a randomized setting to demonstrate how to improve adherence to endocrine treatment. By utilizing the standardized MOATT© approach, adherence rates after 24 weeks could be improved by 12.7 %. Future studies might consider integrating this personal communication approach with other methods, such as digital tools and taking into account patients’ individual characteristics.

## CRediT authorship contribution statement

**Manfred Welslau:** Writing – original draft, Visualization, Investigation, Formal analysis, Conceptualization. **Peter A. Fasching:** Writing – original draft, Investigation, Data curation, Conceptualization. **Nicole Semmler-Lins:** Writing – review & editing, Investigation. **Lothar Mueller:** Writing – review & editing, Investigation. **Erik Belleville:** Writing – review & editing, Conceptualization. **Lorenz Rieger:** Writing – review & editing, Investigation. **Sabrina Uhrig:** Writing – review & editing, Data curation. **Mark-Oliver Zahn:** Writing – review & editing, Investigation. **Benno Lex:** Writing – review & editing, Investigation. **Christoph Uleer:** Writing – review & editing, Investigation. **Natalija Deuerling:** Writing – review & editing, Investigation. **Tobias Hesse:** Writing – review & editing, Investigation. **Dagmar Langanke:** Writing – review & editing, Investigation. **Lothar Häberle:** Writing – review & editing, Formal analysis, Conceptualization. **Hans Tesch:** Writing – original draft, Investigation, Formal analysis, Conceptualization.

## Data availability statement

The data generated in this study are available upon reasonable request from the corresponding author.

## Funding

This study was conducted as an investigator-initiated trial and was partially funded by Lilly. The funding entities did not have any influence on the conduct of the study or the content of the manuscript.

## Declaration of competing interest

The author is an Editorial Board Member/Editor-in-Chief/Associate Editor/Guest Editor for this journal and was not involved in the editorial review or the decision to publish this article.

The authors declare the following financial interests/personal relationships which may be considered as potential competing interests:**E.B.** received honoraria from Novartis, Hexal, BMS, Lilly, Pfizer, Roche, MSD, Bayer, Ipsen, Bluebird, B. Braun, and onkowissen.de for consulting, clinical research management, or medical education activities. **P.A.F.** received honoraria from Roche, Novartis, Pfizer, Daiichi Sankyo, Eisai, Merck Sharp & Dohme, AstraZeneca, Hexal, Lilly, SeaGen, Agendia, Gilead Sciences, participated in advisory boards for Roche, Novartis, Pfizer, Daiichi Sankyo, Eisai, Merck Sharp & Dohme, AstraZeneca, Hexal, Pierre Fabre, Seagen, Agendia, Lilly, Gilead Sciences, Mylan, Menarini Group, Veracyte, Guardant Health, received research funding from Novartis, BioNtech, Cepheid, Roche, Pfizer. **L.H.** is a stakeholder in Biomed Statistics GmbH. **T.H.** received speaker honoraria from Roche, AstraZeneca, Pfizer, Novartis, Lilly, GSK, Tesaro, Seagen, Daiichi Sankyo, Eisai, travel support from Roche, AstraZeneca, Pfizer, Novartis, Lilly, GSK, Tesaro, Seagen, Daiichi Sankyo, Eisai and participated in advisory boards for Roche, AstraZeneca, Pfizer, Novartis, Lilly, GSK, Tesaro, Seagen, Daiichi Sankyo, Eisai. **D.L.** received received speaker honoraria from Lilly Deutschland GmbH, Pfizer Pharma GmbH, Roche Pharma GmbH, Daiichi Sankyo GmbH, Astra Zeneca GmbH, Gilead Science, travel support from Gilead Sciences, Pfizer Pharma GmbH and participated in advisory boards for Roche Pharma GmbH, Lilly Deutschland GmbH, Gilead Sciences, and has stock/stock options from CHOP GmbH, Care and Coach GmbH and Vision Med GmbH. **L.M.** received received travel support from octapharm, Pierre Fabre and participated in advisory boards for Roche. **H.T.** received consulting fees from Roche, Pfizer, AstraZeneca, Daiichi Sankyo, Gilead Sciences, speaker honoraria from Gilead Sciences, Eickeler, Daiichi Sankyo, Novartis, Roche, AstraZeneca, Lilly, MSD, Pfizer, Seagen. **C.U.** received speaker honoraria from RG GmbH für Information Organisation, Friesland Kliniken - St. Johannes Hospital, Medi-Semina GmbH, NIO Kongress GmbH, travel support from ConMed GmbH, participated in advisory boards for Lilly GmbH, Daiichi Sankyo Deutschland GmbH, Exact Sciences Deutschland GmbH, Berufsverband der Urologen eV, Eisai GmbH. **M.W.** received consulting fees from Novartis, CinSol GmbH & Co. KG, received speaker honoraria from AstraZeneca, BMS, Sanofi, SOBI, Novartis, Gilead, Daiichi Sankyo, Janssen, and participated in advisory boards for AstraZeneca, BMS, Sanofi, SOBI, Novartis, Gilead, Daiichi Sankyo, Janssen. **M.O.Z.** received speaker honoraria from Iomedico, AstraZeneca, Gilead, Novartis, and owns stock/stock options of Bayer and Gilead. All other authors (**N.D., B.L., L.R., N.S.-L., S.U.**) declare that they do not have a conflict of interest.
