# Supplementary material for: Standardized patient coaching improves therapy persistence in patients with hormone receptor–positive, HER2–negative advanced/metastatic breast cancer treated with abemaciclib
Source: Breast. 2025 Dec 23;85:104684. doi: 10.1016/j.breast.2025.104684 (PMC12828806; doi:10.1016/j.breast.2025.104684)

**Supplement**

**Supplementary Tables and Figures**

**Table S1:** Inclusion and exclusion criteria of the IMPACT study

| **Inclusion criteria** |
| --- |
| 1. Adult breast cancer patients (age ≥18 years). |
| 1. Patients diagnosed with HR+, HER2− advanced or metastatic breast cancer, confirmed by clinical measures such as standard imaging, and whose disease has progressed after hormonal therapy. The disease must not be amenable to resection with curative intent. |
| 1. Patients receiving abemaciclib treatment in accordance with the SmPC and medical practices of each participating center. |
| 1. Written and signed informed consent was obtained prior to the commencement of study documentation. |
| 1. Patients who have previously received chemotherapy must have recovered from the acute effects of chemotherapy (CTCAE Grade ≤1), except for residual alopecia or Grade 2 peripheral neuropathy, before randomization. A washout period of at least 21 days is required between the last chemotherapy dose and randomization (unless the patient also received radiotherapy during this period). |
| 1. Patients who received adjuvant radiotherapy must have completed it and fully recovered from its acute effects. A washout period of at least 14 days is required between the end of radiotherapy and randomization. |
| 1. The patient must be able to swallow oral medications. |
| 1. Adequate organ function, defined as follows: |
| - Absolute neutrophil count (ANC) ≥1.5 × 10^9^/L |
| - Platelets ≥100 × 10^9^/L |
| - Hemoglobin ≥8 g/dL (patients may receive erythrocyte transfusions to achieve this level, provided that the initial treatment does not begin earlier than the day following the transfusion) |
| - Total bilirubin ≤1.5 times the upper limit of normal (ULN) |
| - Alanine aminotransferase (ALT)/aspartate aminotransferase (AST) ≤3 times ULN |
| - Serum creatinine ≤1.5 times ULN |
| **Exclusion criteria** |
| 1. Patients with serious preexisting medical conditions that would preclude participation in this study, including interstitial lung disease, severe dyspnea at rest or requiring oxygen therapy, history of major surgical resection involving the stomach or small bowel, or preexisting Crohn’s disease or ulcerative colitis, or a chronic condition resulting in baseline Grade 2 or higher diarrhea. |
| 1. Patients experiencing a visceral crisis, as there is currently no data concerning the efficacy and safety of abemaciclib for this patient population. |
| 1. Patients with active bacterial infections requiring intravenous (IV) antibiotics at the time of initiating study treatment, fungal infections, or detectable viral infections such as known human immunodeficiency virus positivity or known active hepatitis B or C (e.g., hepatitis B surface antigen positive). Screening for these conditions is not required for enrollment. |
| 1. Patients with a personal history in the previous 5 years of any of the following conditions: syncope of cardiovascular etiology, ventricular arrhythmia of pathological origin (including, but not limited to, ventricular tachycardia and ventricular fibrillation), or sudden cardiac arrest. |
| 1. Patients with rare hereditary problems of galactose intolerance, total lactase deficiency, or glucose-galactose malabsorption should not take this medication. |
| 1. Patients with contraindications against abemaciclib according to the respective SmPCs. |
| 1. Female patients of childbearing potential must have a negative serum pregnancy test within 7 days prior to receiving the first dose of abemaciclib and agree to use highly effective contraception methods during the treatment period and for 3 weeks after the last dose of abemaciclib. |
| 1. Patients who are not eligible for observation due to severe comorbidities not specified above or deemed unavailable by the treating physician. |

**Table S2**: Clinical trial sites

| **Trial site name** |
| --- |
| Ansbach - ANregiomed gKU Klinikum Ansbach |
| Aschaffenburg - Klinik - Hämato-Onkologische Schwerpunktpraxis |
| Berlin - Praxisklinik - Brustzentrum |
| Bremen - Praxis - Hämato-Onkologische Schwerpunktpraxis |
| Erlangen - Universitätsklinik - Frauenklinik |
| Frankfurt - Onkologie Bethanien - Centrum für Hämatologie |
| Goslar - Praxis - Onkologische Kooperation Harz |
| Hanau - Onkologisches Zentrum - Praxis |
| Hildesheim - Praxisgemeinschaft - Frauenärzte am Bahnhofsplatz |
| Konstanz - Gesundheitsverbund Landkreis Konstanz - Frauenheilkunde |
| Kulmbach - Klinikum - Brustzentrum |
| Landshut - VK & K - Studien GbR |
| Leer - Studienzentrum UnterEms - Studienzentrum |
| Leipzig - St. Elisabeth-Krankenhaus - Brustzentrum |
| Markredwitz - Klinikum Fichtelgebirge - Brustzentrum Frauenklinik |
| Neustadt - Marienhaus Klinikum Hetzelstift - Brustzentrum |
| Neustadt am Rübenberge - Schwerpunktpraxis - Hämatologie/Onkologie |
| Osnabrück - Niels-Stensen-Kliniken GmbH - Brustzentrum Osnabrück |
| Ravensburg - Gemeinschaftspraxis - Hämatologie und Onkologie |
| Rodgau - Gynäkologische Praxis Dr. Rotmann |
| Rotenburg - Agaplesion Diakonieklinikum - Gynäkologisch-Onkologisches Zentrum |
| Stendal - Johanniter-Krankenhaus - Brustzentrum |
| Tübingen - Universitätsklinikum Tübingen - Departement für Frauengesundheit |
| Wetzlar - Lahn-Dill-Kliniken GmbH |
| Wiesbaden - Asklepios Paulinen Klinik - Onkologische Tagesklinik |

**Figure S1**: Study design of the IMPACT study


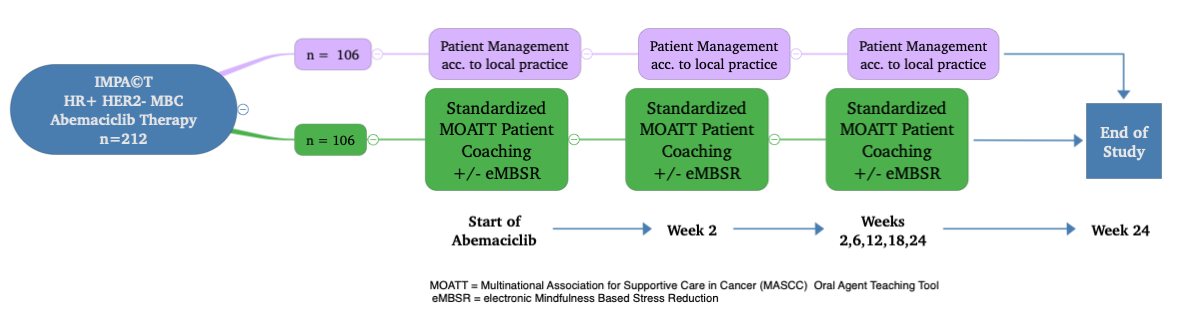

Supplement: Multimedia component 1 [file mmc1.docx]
